# Supplementary material for: The effect of intrapartum prolonged oxygen exposure on fetal metabolic status: secondary analysis from a randomized controlled trial
Source: Front Endocrinol (Lausanne). 2023 Jun 27;14:1204956. doi: 10.3389/fendo.2023.1204956 (PMC10335765; doi:10.3389/fendo.2023.1204956)
Supplement: Supplementary file 3 [file DataSheet_3.zip › Result-X101SC21092977-Z01-J001-B1-42/5.MetKeggEnrichment/O.vs.A/O.vs.A_neg.KEGG_Enrich.scatterplot.pdf]

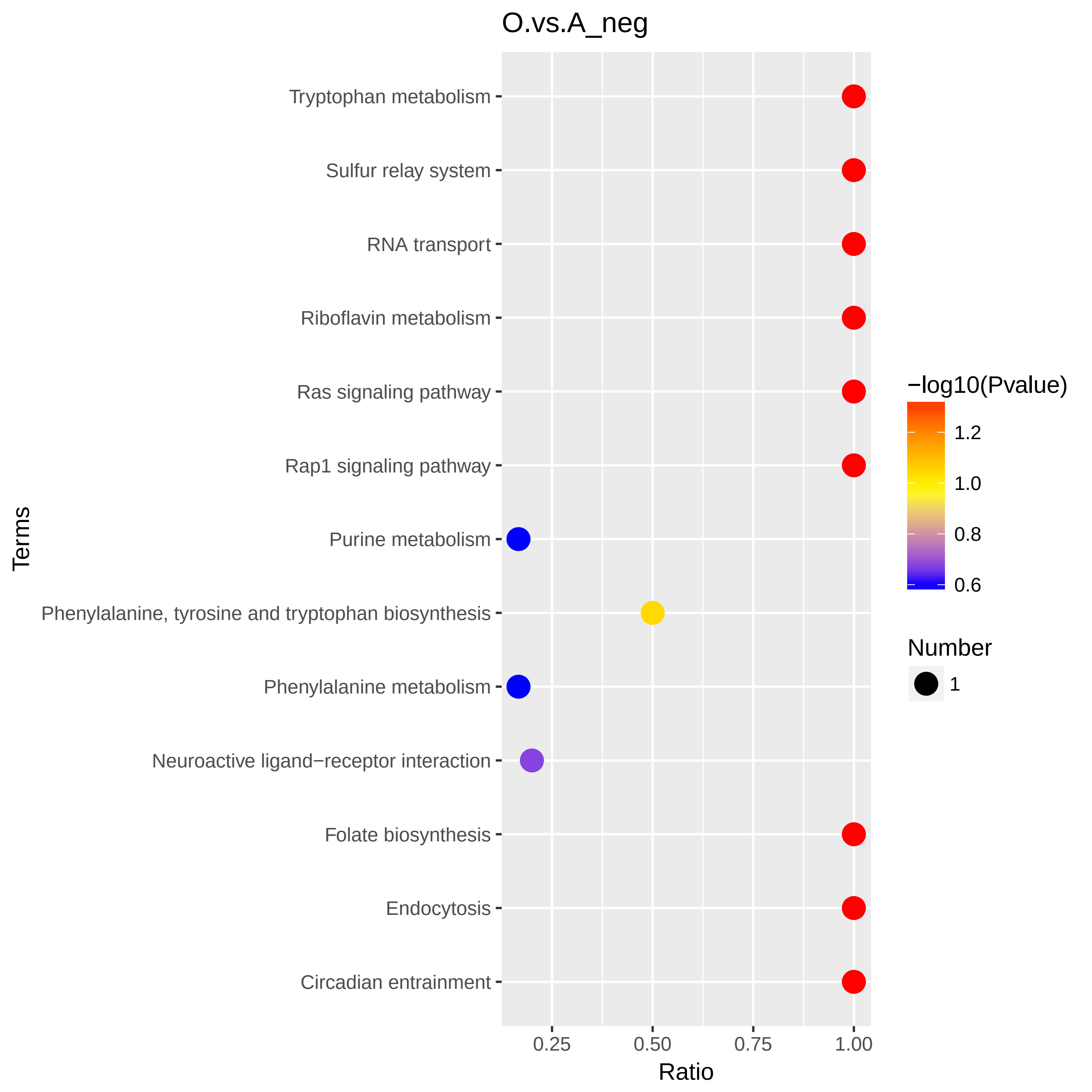
O.vs.A_neg
Tryptophan metabolism
Sulfur relay system
RNA transport
Riboflavin metabolism
Ras signaling pathway
Rap1 signaling pathway
Purine metabolism
Phenylalanine, tyrosine and tryptophan biosynthesis
Phenylalanine metabolism
Neuroactive ligand−receptor interaction
Folate biosynthesis
Endocytosis
Circadian entrainment
Terms
0.25
0.50
0.75
1.00
Ratio
−log10(Pvalue)
1.2
1.0
0.8
0.6
Number
1
